# Role of antibiotic prophylaxis on surgical site infection prevention in a low-risk population undergoing laparoscopic cholecystectomy: A randomized controlled study

**DOI:** 10.1016/j.amsu.2022.103804

**Published:** 2022-05-18

**Authors:** Kaleem Ullah, Abdul Wahab Dogar, ZakaUllah Jan, Hafiz Bilal, Muhammad Junaid Tahir, Ameer Hamza, Muhammad Sohaib Asghar, Zohaib Yousuf

**Affiliations:** a Pir Abdul Qadir Shah Jillani Institute of Medical Sciences, Gambat, Pakistan; b Khyber Teaching Hospital, Peshawar, Pakistan; c Lahore General Hospital, Lahore, 54000, Pakistan; d Dow University of Health Sciences, Ojha Campus, Karachi, Pakistan; e Hamad Medical Corporation, Doha, Qatar

**Keywords:** Efficacy, Surgery, Laparoscopy, Infection, Pathogens, Antibiogram

## Abstract

**Objective:**

To compare the incidence of surgical site infections (SSIs) in low-risk patients undergoing laparoscopic cholecystectomy (LC) with pre-operative antibiotics versus no pre-operative antibiotics administration.

**Study design:**

Randomized controlled study.

**Setting:**

Hepatobiliary department, Pir Abdul Qadir Shah Jeelani Institute of Medical Sciences, Pakistan, from Jul 1, 2018, to Jun 30, 2021.

**Methods:**

This is a prospective, open-label, randomized study. Individuals scheduled for laparoscopic cholecystectomy who met the inclusion requirements were randomly assigned to two groups. Group A patients received pre-operative antibiotics (intravenous cefazolin 2-g), and group B patients were operated on without administration of pre-operative antibiotics. Post-operatively, patients were studied for the occurrence of SSIs for 30 days.

**Results:**

The mean age of patients in group A was 40.6 + 5.2 years, while group B was 41.04 + 5.03. The male to female ratio was 1:3. Gender distribution showed female dominance in both groups, i.e., 78.74% in group A and 76.80% in group B. The incidence of SSI in group A was 3.98%, while in group B was 4.9% (p-value = 0.584). No statistical significance was found while comparing both groups' age, gender, operative duration, and hospital stay.

**Conclusion:**

This study showed comparable results between both groups, and prophylactic antibiotics have no impact in preventing SSIs. In low-risk individuals undergoing laparoscopic cholecystectomy, the incidence of SSIs is quite low, and prophylactic antibiotics can be avoided.

## Highlights

• One of the postoperative complications following cholecystectomy is surgical site infections (SSIs).
• This study aimed to determine the effectiveness of preoperative antibiotic prophylaxis in preventing SSIs.
• We found that prophylactic antibiotics have no impact in preventing SSIs in low-risk individuals undergoing laparoscopic cholecystectomy.

## 1 Introduction

Cholelithiasis is one of the common surgical issues, with an incidence of 10–15% [1,2]. Although age, gender, ethnicity, geographic location, diet, obesity, diabetes mellitus, and dyslipidemia are already known risk factors, despite it being present in 5%–22% of Western populations [3]. Laparoscopic cholecystectomy (LC) has replaced open cholecystectomy as a gold standard for treating gallstones disease [[4], [5], [6], [7]]. In comparison to open cholecystectomy, LC has a significantly lower rate of post-operative infections, post-operative pain, as well as a quicker return to normal activities [7].

One of the most common postoperative complications following cholecystectomy is surgical site infections (SSIs). SSIs are postoperative wound site infections occurring within 4 weeks of surgery, or within a year in patients with implants. SSI can be superficial involving skin and fascia or deep-seated involving cavities [8]. The reported incidence of SSIs in LC patients is quite low (0.4%–1.1%), occurring primarily at the umbilical port site [9]. SSIs result in increased morbidity, extended hospital stays, and a substantial financial burden on health services. Currently, SSIs are considered the most common healthcare-related infections [8]. SSIs can be prevented by various strategies, including the administration of pre-operative prophylactic antibiotics against the common pathogens involved in SSIs [10,11].

There has been much controversy on the use of pre-operative prophylactic antibiotics in LC. Various studies documented the role of pre-operative prophylactic antibiotics in the prevention of SSIs in LC while others found no benefit of pre-operative prophylactic antibiotics in SSIs prevention in low-risk patients [[12], [13], [14]]. Guidelines published by the Scottish Intercollegiate Guidelines Network and American Society of Health-System Pharmacists on SSIs prevention also do not advocate the use of prophylactic antibiotics in low-risk patients undergoing LC [15,16]. Matsui et al. claimed under-reporting of SSIs in literature. They recommended the use of prophylactic antibiotics before LC [17]. A meta-analysis concluded that the pre-operative use of prophylactic antibiotics has a role in reducing the incidence of SSI in LC [18].

The local antibiogram and microbial flora vary across the globe. The incidence of SSIs can vary from population to population. Most of the available data is from developed countries. This study aimed to determine the effectiveness of preoperative antibiotic prophylaxis in preventing SSIs in low-risk LC patients in the Pakistani population.

## 2 Methods

This prospective, open-label, randomized, controlled study was conducted at the hepato-biliary department, Pir Abdul Qadir Shah Jeelani Institute of Medical Sciences, Gambat, Pakistan, from 1st July 2018 to 30th June 2021.

### 2.1 Inclusion criteria

1. Symptomatic cholelithiasis
2. Any gender
3. 18–60 years old
4. ASA score I/II

### 2.2 Exclusion criteria

1. Asymptomatic cholelithiasis
2. ASA score III or more
3. Comorbidities like diabetes mellitus, malignancy, history of steroids, and immune suppression use
4. Pregnancy
5. Laparoscopic cholecystectomy converted to open cholecystectomy

All patients meeting the inclusion criteria were randomly allocated in two groups using the lottery method. Group A (antibiotic group) patients were administered a single dose of pre-operative antibiotic (injection cefazolin 2 gm I.V. 30 min before incision). Group B (no-antibiotic group) patients were not given any prophylactic antibiotic pre-operatively. Postoperatively none of the groups were given any intravenous or oral antibiotics. The study was registered with the registry of Pir Abdul Qadir Shah Jeelani Institute of Medical Sciences (Unique identification no: PASQJIMS/IRB/649). Patients who met the eligibility criteria were admitted through the outpatient department. All participants were given a thorough explanation of the study's purpose and benefits followed by verbal and written informed consent.

A detailed history and clinical examination followed by baseline investigations like CBC, chest x-ray, liver function tests, ECG, serum creatinine, and ultrasound abdomen especially for the biliary tract was performed. Standard four ports laparoscopic cholecystectomy was performed in all the patients. “Critical view of safety” was considered a standard operating technique for all the patients. Gall bladder specimen was retrieved in an endo-bag through the umbilical port and was sent for histopathology. The routine sub-hepatic drain was not placed, however, whenever the need was felt, drain was placed. All the port incisions were stitched with prolene 3/0 suture. Consultant board-certified surgeons did all surgeries. Patients were discharged in stable conditions on the next day of the procedure and no antibiotic was advised for home medication.

**Fig. 1:**
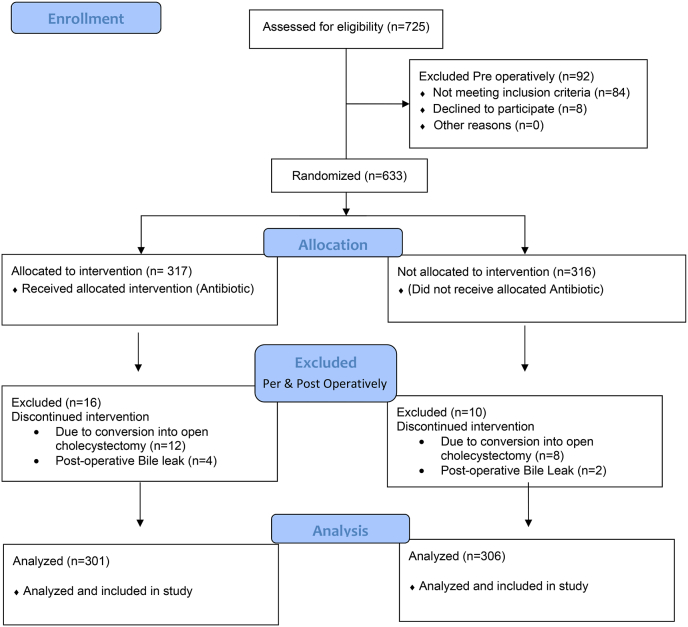
Consort flow diagram of study's patient selection criteria.

A total of 725 patients were assessed for eligibility into the study and underwent cholecystectomy. 92 patients were excluded from the study preoperatively, 08 patients refused to get enrolled into the study, 62 patients had acute cholecystitis, 24 patients turned out to be diabetic and 06 patients were pregnant females and were not included in this study. Rest 633 patients were randomized into two groups labeled as ‘‘antibiotic group’’ (n = 317) and no antibiotic group (n = 316). All the 633 patients of both groups were operated on (standard laparoscopic cholecystectomy) and a total of 20 patients dropped out of the study due to conversion into open cholecystectomy, 12 from the antibiotic group (n = 305) and 8 from no antibiotic group (n = 308). The conversion from laparoscopic to open cholecystectomy was done due to excessive bleeding from cystic artery/liver bed which couldn't be controlled laparoscopically (n = 3), difficult anatomy resulting in not clear visualization of the biliary tree (n = 6), and difficult mobilization of the gall bladder due to acute cholecystitis resulting in mucocele, empyema gall bladder and massive adhesions of the omentum (n = 11). Postoperatively total of 6 patients dropped out of the study due to bile leakage i.e. 4 from the antibiotic group (n = 301) and 2 from the non-antibiotic group (n = 306) [Fig. 1]. Sampling was done through the non-probability consecutive sampling technique.

Post-surgery all the patients were followed up after one week for port site infection. The SSI assessment was done and recorded in terms of pain, redness, raised temperature, and discharge. In cases of no SSI, sutures were removed on the 7th day and follow-up was only advised in case of any adverse events. Those patients who presented with SSI were followed up weekly until the infection resolved. A wound swab was sent for the culture and sensitivity of every SSI. Management of wound care was provided to affected patients: including dressing, antibiotics, and drainage as needed. Intention to treat protocol was used to tackle the loss to follow up within groups.

Different variables including age, gender, body mass index (BMI), hospital stay, and wound status were recorded on the pre-designed Proforma. Primary outcomes were incidence of SSI among both groups. Data analysis was done using SPSS version 22.0. Mean ± Standard deviation was calculated for quantitative variables like age, BMI, duration of surgery, and hospital stay. Frequencies and percentages were calculated for categorical variables like gender, biliary spillage, and wound infection. All the results were presented in tabular and descriptive form. A Chi-square test and *t*-test were applied to test the statistical significance for the incidence of SSIs in both groups. A P-value of less than 0.05 was considered significant.

## 3 Results

A total of 607 patients were included in the study. The mean age overall was 40.81 + 5.11 years. The overall male to female ratio was 1:3, 136(22.24%) patients were males, and 472(77.76%) were females. The overall incidence of SSI was 4.45%.

The mean age of patients in group A was 40.60 + 5.21 years, while in group B the mean age was 41.04 + 5.03 years. In group A, 237(78.74%), while in group B, 235(76.80%) of patients were females. Mean BMI in group A was 26.38 + 1.807 kg/m^2^, while group B was 26.49 + 4.058 kg/m2. No statistically significant difference was noted while comparing age, gender, BMI, duration of surgery, and hospital stay (Table 1).

**Table 1:** Patient's demographics and operative variables.

Parameters		Group A (n = 301)	Group B (n = 306)	p-value
**Gender**	Male	64(21.26%)	71(23.20%)	0.565
	Female	237(78.74%)	235(76.80%)	
Age (years)		40.60 ± 5.210	41.04 ± 5.034	0.290
BMI (kg/m^2^)		26.38 ± 1.807	26.49 ± 4.058	0.667
Operative time (minutes)		38.71 ± 3.605	39.02 ± 3.535	0.825
Biliary spillage		53(17.60%)	61(19.93%)	0.463
Drain placement		110(30.56%)	118(34.71%)	0.607
Hospital stays (Hours)		26.52 ± 5.691	26.34 ± 5.232	0.685

In Group A, the incidence of SSI was 12(3.98%), while in Group “B,” the incidence of SSIs was 15(4.9%). This comparison was statistically not significant (p-value = 0.584) (Table 2).

**Table 2:** Comparison of SSIs in both groups.

SSIs	Group A (Antibiotic group) n = 301	Group B (Non-Antibiotic group) N = 306	P-value
Erythema	6(1.99%)	8(2.61%)	0.584
Seroma	3(0.99%)	4(1.30%)	
Abscess	3(0.99%)	3(1.17%)	
Total	12(3.98%)	15(4.9%)	

## 4 Discussion

The role of antibiotic prophylaxis in open cholecystectomy in SSIs prevention is well documented via literature [19,20]. But its role in minimally invasive surgery i.e., LC is controversial [21]. The guidelines on SSIs prevention published by the Scottish Intercollegiate Guidelines Network and the American Society of Health-System Pharmacists do not advocate prophylactic antibiotics in non-complicated LC cases [15,16]. However, these guidelines are not uniformly followed all over the world and few follow local guidelines or practice on clinical-based experience. Like other national institutes our institute practice the routine administration of pre-operative antibiotics (injection ceftriaxone 1 gm IV) in all adult patients undergoing LC.

The administration of prophylactic antibiotics ideally should prevent postoperative infections, thus reducing the morbidity and health care costs associated with the management of SSIs [10,22]. However, there is also a global campaign to minimize the inappropriate use of antibiotics to reduce antibiotics resistance and associated costs [23,24].

In the current study, we included low-risk patients who underwent LC for symptomatic cholelithiasis. In another study majority of the patients who underwent LC were females. Also, the mean BMI of groups was more >26 kg/m^2^, highlighting the disease prevalence in female and overweight patients [25].

We noticed a comparable incidence of SSIs in both groups and antibiotic prophylaxis was not found to be much effective in these low-risk patients undergoing LC. The possible reasons might be good patients i.e., low-risk patients, and minimally invasive surgery.

In our study, the overall incidence of SSI was 4%. SSI incidence was 3.33% in patients administered pre-operative antibiotics, while the SSIs incidence was 4.7% in cases without antibiotic administration. There was a slightly higher incidence of SSIs in the non-antibiotic group, which was statistically not significant. Our study highlights that antibiotic prophylaxis in low-risk LC does not affect the incidence of SSIs.

Similarly, another study reported SSIs incidence of 1.79% (5 out of 279 patients) in the antibiotic group and 1.56% incidence (3 out of 192 cases) in the non-antibiotic group [25]. Although their study reported a slightly lower incidence of SSIs in both groups in comparison to our study. However, there was a comparable incidence of SSIs in both groups like our study and antibiotic prophylaxis did not show an effective role in reducing the incidence of SSIs.

The randomized controlled trial by Jay Narayan Shah et al. [23] also showed no significant difference in the incidence of SSIs between antibiotics and non-antibiotics group (p = 0.442). They reported 4.8% of the overall incidence of SSIs which was almost similar to the overall incidence of SSIs reported in this study (4%).

On other hand, Matsui et al. [17] found that the incidence of SSIs was significantly higher in the non-antibiotics group than in the antibiotics group (3.7% vs. 0.8% with p-value = 0.001). And they strongly advocated antibiotic prophylaxis in LC patients even in low-risk patients. The findings of this trial are contrary to our study, might be due to different patient populations and different study settings. A non-randomized study regulated by Farello and Cerofolini also gave contrasting results to our study [26].

Based on this study and other previous similar studies discussed above, we adopted the policy of no routine pre-operative antibiotic prophylaxis in low-risk LC cases. This study was conducted in a local scenario with different social and patient backgrounds. Hopefully, this study will benefit similar institutions in the country.

The limitation of our study was that we did not include acute cholecystitis cases. Our study findings cannot be generalized to all cases undergoing LC for acute cholecystitis as we excluded anyone with a high ASA score and pregnant females. These patients will require a separate study proposal with a different set of protocols. Other than that, the classification of SSI used (erythema, seroma and abscess) may be non-reproducible. Hence, it is difficult to make such an interpretation without a multicentric study with high evidential value.

## 5 Conclusion

This study showed that SSIs have a low incidence in LC. We did not find a role of prophylactic antibiotics in preventing SSIs in low-risk patients undergoing LC. Therefore, antibiotics can be avoided in low-risk patients undergoing LC.

## Provenance and peer review

Not commissioned, externally peer reviewed.

## Sources of funding

None.

## Author contributions

K·U, Z.U, and A.W.D conceived the idea; K·U, Z.U, H·B, and A.W.D collected the data; A.H and M.S.A analyzed and interpreted the data; K·U, M.J.T, H·B, M.S.A, and Z.Y did write up of the manuscript; and finally Z.Y, M.S.A, M.J.T, and A.W.D reviewed the manuscript for intellectual content critically. All authors approved the final version of the manuscript.

## Ethics statement

All ethical requirements were fulfilled before commencement of study.

## Funding

None.

## Consent

All participants were given a thorough explanation of the study's purpose and benefits followed by verbal and written informed consent.

## Registration of research studies

Name of the registry: Pir Abdul Qadir Shah Jeelani Institute of Medical Sciences.
Unique Identifying number or registration ID: PASQJIMS/IRB/649.
Hyperlink to your specific registration (must be publicly accessible and will be checked):

## Guarantor

Muhammad Sohaib Asghar.

## Declaration of competing interest

The authors declare that there is no conflict of Interest.
